# Positive Association Between Body Mass Index and the Likelihood of Reporting an Overall Cancer Diagnosis Among College Students in the United States

**DOI:** 10.1002/cam4.71508

**Published:** 2025-12-30

**Authors:** Shenghui Wu, Martie Thompson, Richard W. Christiana, Adam Hege, Jennifer Schroeder Tyson

**Affiliations:** ^1^ Department of Public Health, Beaver College of Health Sciences Appalachian State University Boone North Carolina USA

**Keywords:** body mass index, cancer, college students, obesity, overweight

## Abstract

**Introduction:**

We conducted the first epidemiologic study to examine the dose–response relationship between body mass index (BMI) and the likelihood of reporting an overall cancer diagnosis, along with the association between overweight or obese and cancer diagnosis among college students in the United States.

**Methods:**

The American College Health Association‐National College Health Assessment (ACHA‐NCHA) self‐reported, cross‐sectional data on demographic information, physical activity, BMI, smoking status, and overall cancer from 2019 to 2022 (*n* = 275,185; 0.08% cancer cases) were used. A cubic spline model and logistic regression analyses were performed to evaluate associations between BMI and the likelihood of reporting a cancer diagnosis, adjusting for relevant covariates.

**Results:**

The cubic spline observed that higher BMI (kg/m^2^) was associated with a greater likelihood of reporting an overall cancer diagnosis after adjusting for age, sex, race, ethnicity, education level, physical activity, and smoking status (*p* for linear relation = 0.02 and *p* for overall association < 0.0001). Specifically, each 1 kg/m^2^ increase in BMI was associated with a 1% increase in the odds of a cancer diagnosis. Multivariable‐adjusted logistic regression analyses revealed that both overweight (30 > BMI ≥ 25 kg/m^2^) [odds ratio (OR): 1.20 (95% confidence interval (CI): 1.08–1.34)], and obesity (BMI ≥ 30 kg/m^2^) [1.48 (1.32–1.65)] were positively associated with a cancer diagnosis.

**Conclusion:**

Higher BMI, especially overweight/obesity, was associated with a greater likelihood of reporting a cancer diagnosis among college students in the United States. To prevent and control cancer, targeted interventions aimed at maintaining a healthy weight in this population are warranted.

## Graphical abstract

Higher body mass index, particularly overweight/obesity, was associated with a greater likelihood of reporting an overall cancer diagnosis among college students in the United States. To prevent and control cancer, targeted interventions aimed at promoting and maintaining a healthy weight in this population are warranted.

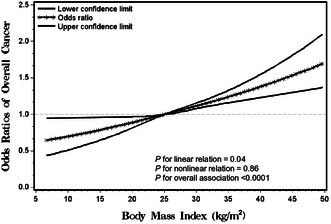

## 1 Introduction

In 2023, it was estimated that more than 1.9 million new cancer cases would be diagnosed and 609,820 people would die from cancer in the United States (U.S.) [1]. Overweight (defined as a body‐mass index [BMI] between 25 and 29.9 kg/m^2^) and obesity (BMI ≥ 30 kg/m^2^) are important risk factors for several common cancer types, including breast, colon, and endometrial cancers [2, 3]. The growing enrollment of college students in the U.S. [4]. represents an increasingly important segment of the U.S. population. Therefore, research focused on understanding or addressing health behaviors of college students in this group, such as obesity, is essential. The overweight/obese prevalence rate is high among U.S. undergraduate college students (37.3% in females and 34.1% in males) [5]. Global cohort studies and meta‐analyses have identified positive associations between BMI, obesity, and cancer risk in young adults. However, these findings are constrained by limitations such as missing information in the studies, inadequate data, insufficient power from individual studies, and unrepresentative samples [6, 7, 8, 9]. The mechanisms linking excess weight to cancer risk are not fully understood. Locally, adipose tissue inflammation and related alterations in the microenvironment may be associated with tumor promotion; systemically, adipose inflammation‐related circulating metabolic and inflammatory mediators (such as dyslipidemia and insulin resistance) may also play a role in the tumor‐promoting effects of obesity [10, 11]. Conversely [12, 13], certain cancer treatments, including chemotherapy, steroid treatment, and hormone therapies, have been associated with weight gain [13, 14, 15, 16, 17], and cancer itself might cause obesity after cancer development, although the mechanisms are unclear [13, 14, 15, 16, 17]. To our knowledge, no epidemiological studies have examined the association between BMI and cancer risk among U.S. college students [6, 7, 8, 9]. To address this gap, we investigated the dose–response relation between BMI and cancer, along with the associations between overweight/obesity and the likelihood of reporting an overall cancer diagnosis. These analyses were conducted using data from the American College Health Association‐National College Health Assessment (ACHA‐NCHA).

## 2 Materials and Methods

### 2.1 Data Source and Sample

The ACHA‐NCHA is a nationwide survey of college students that collects a wide range of health and health‐related behaviors and information. We used data from the 2019 to 2022 administration of the ACHA‐NCHAIII, which captured cross‐sectional, web‐based, self‐reported data from students attending U.S. institutions of higher education. Participating institutions self‐selected to administer the survey to a randomly selected sample of students aged 18 years and older. This study was exempt from Appalachian State University Institutional Review Board review because the data were de‐identified.

### 2.2 Exposure and Outcome Measurements

The information gathered included demographic characteristics such as age, sex, race, ethnicity, health insurance status, education level, tobacco or nicotine products ever used, weight, height, and moderate and vigorous physical activity (PA) (total minutes). BMI was calculated as weight in kilograms divided by height in meters squared (kg/m^2^). Participants were categorized as normal weight (< 25 kg/m^2^), overweight (25–29.9 kg/m^2^), or obese (≥ 30 kg/m^2^) using standard cutoffs [18]. Meeting U.S. aerobic PA guidelines was defined as engaging in either PA ≥ 150 min/week of moderate aerobic or PA ≥ 75 min/week of vigorous activity [19]. Food insecurity was measured using the United States Department of Agriculture Food Security 6‐item Short Scale [1, 2, 3, 4, 5, 6], with participants classified into three categories based on their scores: very low food security [5, 6], low food security [2, 3, 4], and high or marginal food security (0–1). Cancer diagnosis was identified through self‐report, based on whether participants had ever been informed by a healthcare provider that they had cancer. The study focused exclusively on overall cancer diagnosis and did not include information on specific cancer types due to unavailability.

### 2.3 Data Analysis

The *t*‐tests were used to compare continuous participant characteristics, and chi‐square tests were applied to categorical variables. To evaluate the dose–response relationship between BMI and the likelihood of reporting an overall cancer diagnosis, restricted cubic spline logistic regression analysis was employed to estimate the odds ratios (ORs) associated with continuous BMI (kg/m^2^) [20]. Extreme BMI values (< 6.7 kg/m^2^) [21] were excluded to reduce the influence of outliers. Knots were placed at the 5th, 50th, and 95th percentiles of the BMI distribution. Logistic regression models were used to calculate univariate and multivariable‐adjusted ORs with 95% confidence intervals (CIs) for the associations between overweight/obesity and the likelihood of an overall cancer diagnosis. Multivariable models adjusted for demographic information and potential cancer risk or protective factors, including age, sex, race, ethnicity, education, PA, and smoking status, regardless of their statistical significance in univariate analyses. A *p* less than 0.05 was considered statistically significant. Additional interaction analyses were performed to evaluate whether the associations varied across subgroups, thereby testing the consistency of the findings. All statistical analyses were conducted using SAS version 9.4 (Cary, NC, USA).

## 3 Results

The average age was 23.06 years (SD = 6.6) for 275,185 participants with available cancer and BMI data; most students (79.97%) were 25 years or younger and 67.66% were female (Table 1). Overall, 38.74% of the participants fell into the overweight/obese category. Among all students, 2277 (0.08%) reported a cancer diagnosis (Table 1). Compared with students without cancer, those with cancer were more likely to be older, female, White, non‐Hispanic, undergraduate students, cigarette smokers, have higher BMIs, be classified as overweight/obese, and less likely to engage in sufficient PA according to national recommendations (all *p* < 0.05).

**TABLE 1:** Characteristics stratified by overall cancer status: The American College Health Association National College Health Assessment (2019–2022).

Characteristics	Cancer status (number, percentage)			*p*
	Yes (*n* = 2277; 0.83%)	No (*n* = 272,908; 99.17%)	Total (*n* = 275,185)	
**Demographics**				
Age (years)^a^	Mean: 33.58 SD: 15.2	Mean: 22.97 SD: 6.4	Mean: 23.06 SD: 6.6	< 0.0001
≤ 25	1123 (46.08)	230,022 (80.26)	231,145	< 0.0001
> 25	1314 (53.92)	56,563 (19.74)	57,877	
Gender				0.02
Female	1593 (69.96)	184,596 (67.64)	186,189	
Male	684 (30.04)	88,312 (32.36)	88,996	
Race				< 0.0001
White	1685 (75.83)	174,437 (68.00)	176,122	
Asian	205 (9.23)	46,120 (17.98)	46,325	
Black	114 (5.13)	16,911 (6.59)	17,025	
American Indian or Native Alaskan	98 (4.41)	5941 (2.32)	6039	
Others	120 (5.40)	13,123 (5.12)	13,243	
Ethnicity				
Hispanic	316 (12.97)	43,164 (15.06)	43,480	0.004
Non‐Hispanic	2121 (87.03)	243,421 (84.94)	245,542	
Education				
Undergraduate	1418 (58.28)	218,298 (76.21)	219,716	< 0.0001
Master's and above degrees	892 (36.66)	64,387 (22.48)	65,279	
Other	123 (5.06)	3762 (1.31)	3885	
Insurance				0.54
Yes	2337 (97.05)	271,631 (96.83)	273,968	
No	71 (2.95)	8880 (2.95)	8951	
**Lifestyle factors**				
Body mass index (kg/m^2^)	Mean: 26.77 SD: 7.5	Mean: 24.99 SD: 5.8	Mean: 25.002 SD: 5.8	< 0.0001
Normal (< 25)	1186 (48.67)	175,856 (61.36)	177,042	< 0.0001
Overweight (25 to < 30)	634 (26.02)	66,014 (23.03)	66,648	
Obese (≥ 30)	617 (25.32)	44,715 (15.60)	45,332	
Ever smokers				
Yes	947 (38.94)	95,806 (33.48)	96,753	< 0.0001
No	1485 (61.06)	190,319 (66.52)	191,804	
Met the US guidelines for only aerobic PA for adults^b^				
Yes	1526 (63.29)	193,321 (68.20)	194,847	< 0.0001
No	885 (36.71)	90,154 (31.802)	91,039	

Abbreviation: PA, physical activity.

^a^ All participants were at least 18 years of age at enrollment.

^b^ ≥ 150 min/week of moderate aerobic PA or ≥ 75 min of vigorous PA.

**FIGURE 1:**
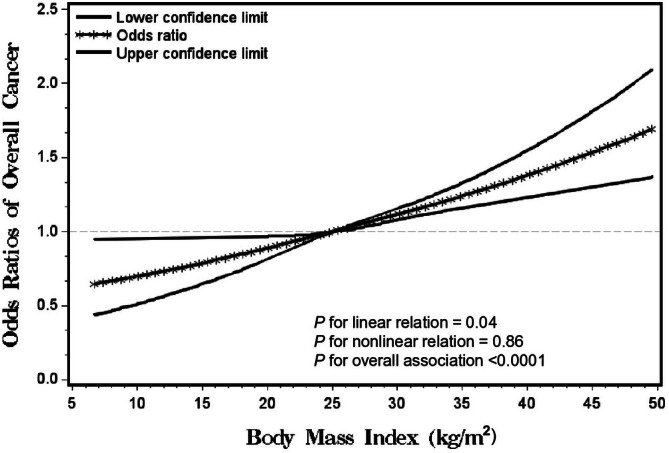
Smoothed plot for odds ratios (ORs) of the overall cancer diagnosis according to body mass index (BMI) (kg/m^2^). The ORs were estimated by using the restricted cubic‐spline logistic regression models with knots placed at the 5th, 50th, and 95th percentiles of BMI. The model was adjusted for age, sex, race, ethnicity, education, physical activity, and smoking status.

Figure 1 illustrates the dose–response relationship between BMI and the likelihood of reporting an overall cancer diagnosis, based on a restricted cubic spline model adjusted for age, sex, race, ethnicity, education, PA, and smoking status. Higher BMI was associated with a greater likelihood of reporting a cancer diagnosis (*p* for overall relation = 0.04, *p* for nonlinear relation = 0.86, and *p* for linear relation < 0.0001). The statistically significant linear trend indicates that each one‐unit (kg/m^2^) increase in BMI was associated with a 2% increase in the odds of reporting a cancer diagnosis [OR: 1.02 (95% CI: 1.001–1.05)]. The findings align with the multivariable‐adjusted association between continuous BMI and overall cancer diagnosis reported in **Table** **2** [OR: 1.022 (95% CI: 1.016–1.029)].

**TABLE 2:** Associations between body mass index and overall cancer: The American College Health Association National College Health Assessment (2019–2022).

	Univariate Analysis		Multivariable adjusted Analysis^a^	
	OR (95% CI)	*p*	OR (95% CI)	*p*
Body Mass Index (kg/m^2^)	1.04 (1.036–1.05)	< 0.0001	1.022 (1.016–1.029)	< 0.0001
Overweight vs. Normal weight^b^	1.42 (1.29–1.57)	< 0.0001	1.20 (1.08–1.34)	0.0008
Obesity^c^ vs. Normal weight	2.05 (1.86–2.26)	< 0.0001	1.48 (1.32–1.65)	< 0.0001

Abbreviations: OR, Odds ratios; CI, Confidence intervals.

^a^ Adjusted for age, sex, race, ethnicity, education, physical activity, and smoking status.

^b^ Normal weight: Body mass index < 25 kg/m^2^; Overweight: 25 kg/m^2^ ≤ Body mass index < 30 kg/m^2^.

^c^ Obese: Body mass index ≥ 30 kg/m^2^.

Overweight (25–29.9 kg/m^2^) was positively associated with a 42% higher likelihood of reporting a cancer diagnosis in unadjusted models (OR: 1.42; 95% CI: 1.29–1.57), and a 20% increase (OR: 1.20; 95% CI: 1.08–1.34) after adjusting for age, sex, race, ethnicity, education, PA, and smoking status versus normal weight (**Table** **2**). Obesity (≥ 30 kg/m^2^) was associated with a 105% higher likelihood of reporting a cancer diagnosis in unadjusted models (OR: 2.05; 95% CI: 1.86–2.26) and a 48% increase (OR: 1.48; 95% CI: 1.32–1.65) in adjusted models. No statistically significant interactions were observed. Results remained consistent after further adjustment for food security.

## 4 Discussion

Using data from a large sample of college students across the U.S., higher BMI was associated with a greater likelihood of reporting a cancer diagnosis; specifically, each 1 kg/m^2^ increase in BMI was linked to a 2% higher likelihood of reporting cancer. After adjusting for age, sex, race, ethnicity, education, PA, and smoking status, overweight and obesity were related to 20% and 48% increases in the likelihood of a cancer diagnosis, respectively. These findings suggest that BMI may have a significant impact on the likelihood of reporting cancer, with evidence of a dose–response relationship in which higher BMI corresponds to a greater likelihood of cancer diagnosis. However, a causal, biologically based dose–response relationship cannot be established due to the cross‐sectional nature of the data and lack of temporality.

To our knowledge, although no prior studies have examined the association between BMI and cancer in U.S. college students, one study involving 1765 college students reported that overweight (BMI 25–29.9 kg/m^2^) and obesity (BMI ≥ 30 kg/m^2^) were significantly associated with lower overall academic achievement, more depressive symptoms, and greater use of diet pills for weight loss (all *p* < 0.05) [22]. That study also found that overweight and obese female students had higher rates of panic disorder, while obese male students reported significantly higher rates of lifetime trichotillomania [22]. These findings highlight the broader psychological and behavioral burden associated with overweight and obesity among college students. Our findings add to this literature by suggesting that obesity was associated with a greater likelihood of cancer diagnosis compared to overweight status. Specifically, obesity appears to be more strongly linked to reporting an overall cancer diagnosis than being overweight. Maintaining a healthy body weight, particularly keeping BMI within the normal range, may help lower the risk of cancer among college students. However, it is important to note that the observed positive association between BMI/obesity and cancer diagnosis could reflect reverse causation in some cases. For example, cancer development or chemotherapy (such as gaining weight due to burning fewer calories), steroid treatment (such as appetite and fat tissue increase due to steroid medicines), and hormone therapy (such as fat increase and muscle loss due to hormone therapy) [13, 14, 15, 16, 17] could result in weight gain if the cancer diagnosis precedes the BMI measurement. Therefore, prospective cohort studies are needed to clarify the temporal relationship between BMI and cancer risk in college students.

Our study has some limitations. Its cross‐sectional design only allows for the identification of associations, not causal relationships. It is therefore difficult to determine whether cancer developed after changes in BMI or if BMI levels were influenced by cancer. Since BMI level was measured only at the time of data collection, we cannot ascertain whether cancer patients lost weight following medical advice or due to the disease itself. It is possible that patients who experienced significant loss represent a smaller proportion of the studied population, or that some cancer patients with weight loss remained classified as overweight/obesity. Additionally, survivor bias may have influenced the results, as cancer patients who did not survive to participate were not included. Prospective cohort studies are needed to better understand the underlying mechanisms and to explore the potential dose–response relationship between BMI and cancer risk across the lifespan. The cancer identification was self‐reported, so the biological confirmation was not possible. However, this self‐reported method has been validated in previous research [23, 24]. The date of cancer diagnosis was not collected, which limits accuracy if cancer was diagnosed long before BMI measurement (e.g., during childhood). Furthermore, information on cancer‐specific sites was not available, preventing analysis of BMI's relationship with cancer types by site, despite the heterogeneity of cancer types and their varying etiologies related to BMI. Geographic location data were also inaccessible, so regional variables could not be assessed. Since participation was voluntary and schools self‐selected into the study, the data may not be fully generalizable to all U.S. college students. Nonetheless, previous research comparing this dataset to nationally representative samples of U.S. college students has demonstrated its validity and reliability in reflecting the broader student population [25]. Finally, residual confounding from unmeasured or inadequately measured covariates cannot be entirely ruled out, although many known covariates were controlled for in analyses.

Our research had several key strengths. It is, to our knowledge, the first epidemiological investigation to explore the relationship between BMI and cancer among U.S. college students. Notably, we identified clear dose–response patterns linking BMI with the likelihood of overall cancer diagnosis; contributing new insights that may support future efforts in cancer prevention and control. By using a large, nationally randomly selected sample, the study minimizes potential biases commonly found in studies based on clinical populations or other non‐randomly selected groups with pre‐existing health conditions. Moreover, the inclusion of a wide array of cancer‐related variables enabled a thorough and multifaceted examination of contributing factors.

In conclusion, increased BMI, particularly overweight and obesity, was significantly linked to a greater likelihood of reporting a cancer diagnosis, even after adjusting for confounders. Consequently, college students with a history of cancer have obesity. These findings highlight the need for targeted interventions to reduce overweight and obesity among college students. Future prospective cohort studies are necessary to establish the temporal link between BMI and cancer risk in this population.

## Author Contributions

**Shenghui Wu:** conceptualization (lead), formal analysis (equal), methodology (equal), project administration (equal), software (lead), supervision (lead), validation (lead), visualization (lead), writing – original draft (lead), writing – review and editing (lead). **Martie Thompson:** data curation (equal), writing – review and editing (equal). **Richard W. Christiana:** writing – review and editing (equal). **Adam Hege:** writing – review and editing (equal). **Jennifer Schroeder Tyson:** writing – review and editing (equal).

## Funding

The authors have nothing to report.

## Ethics Statement

This study was exempt from Appalachian State University Institutional Review Board review because it used de‐identified data.

## Conflicts of Interest

The authors declare no conflicts of interest.

## Data Availability

The data that support the findings of this study are available from the American College Health Association. However, restrictions apply to the availability of these data, which were used under license for the current study and are not publicly accessible. For access inquiries, please contact the American College Health Association using the information provided below: American College Health Association, National College Health Assessment Program Office, 8455 Colesville Road, Suite 740, Silver Spring, MD 20910, P: (410) 859‐1500, F: (410) 859‐1510, https://www.acha.org/.
